# Single Step In Situ Detection of Surface Protein and MicroRNA in Clustered Extracellular Vesicles Using Flow Cytometry

**DOI:** 10.3390/jcm10020319

**Published:** 2021-01-17

**Authors:** Hee Cheol Yang, Won Jong Rhee

**Affiliations:** 1 Department of Bioengineering and Nano-Bioengineering, Incheon National University, Incheon 22012, Korea; aaaa428a@gmail.com; 2 Division of Bioengineering, Incheon National University, Incheon 22012, Korea

**Keywords:** extracellular vesicles, single step in situ detection, extracellular vesicle cluster, molecular beacon, flow cytometer

## Abstract

Because cancers are heterogeneous, it is evident that multiplexed detection is required to achieve disease diagnosis with high accuracy and specificity. Extracellular vesicles (EVs) have been a subject of great interest as sources of novel biomarkers for cancer liquid biopsy. However, EVs are nano-sized particles that are difficult to handle; thus, it is necessary to develop a method that enables efficient and straightforward EV biomarker detection. In the present study, we developed a method for single step in situ detection of EV surface proteins and inner miRNAs simultaneously using a flow cytometer. CD63 antibody and molecular beacon-21 were investigated for multiplexed biomarker detection in normal and cancer EVs. A phospholipid-polymer-phospholipid conjugate was introduced to induce clustering of the EVs analyzed using nanoparticle tracking analysis, which enhanced the detection signals. As a result, the method could detect and distinguish cancer cell-derived EVs using a flow cytometer. Thus, single step in situ detection of multiple EV biomarkers using a flow cytometer can be applied as a simple, labor- and time-saving, non-invasive liquid biopsy for the diagnosis of various diseases, including cancer.

## 1. Introduction

Recently, extracellular vesicles (EVs) have been a subject of great interest as sources of novel biomarkers for liquid biopsy that can overcome the limitations of previously used biomarkers for cancer [1,2,3]. As central mediators of intercellular communication, EVs, including exosomes, play a crucial role in disease pathogenesis, including cancer progression and metastasis [4,5,6,7,8]. High concentrations of EVs can be found in various bodily fluids, including blood [9], urine [10], saliva [11], and breast milk [12]. Unlike other circulating biomarkers, because of their endosomal origin, EVs are enriched in virtually every type of biomolecule, such as proteins and RNA, which are either encapsulated inside or located on the surface of EVs [13,14]. Among EV biomarkers, surface proteins and inner microRNAs (miRNAs) have been extensively studied to develop novel biomarkers for liquid biopsy [15,16]. Because cancers are heterogeneous and a single biomarker cannot accurately reflect the stage of disease progression, it is obvious that multiplexed detection is required to achieve cancer diagnosis with high accuracy and specificity.

The currently developed methods for multiplexed detection of EV proteins and miRNAs are based on the analysis of biomolecules independently and separately, using different methods such as real-time polymerase chain reaction (PCR) [17,18] and enzyme-linked immunosorbent assay [19]. These are time-consuming, expensive, and laborious, and thus, there is a high need for the development of a simple and efficient method for the detection of multiple proteins and miRNAs in a single reaction. We recently developed a technique for simultaneous multiplexed detection of EV surface proteins and miRNAs [20]. In this method, EVs are first captured using antibody-conjugated magnetic beads. The miRNAs inside the EVs are then detected using molecular beacons (MBs) and nano-sized oligonucleotide probes [20,21,22,23], while EV surface proteins, including CD63, are detected using fluorescence dye-labeled antibodies. The method uses a fluorometer to quantify the fluorescent intensities of the miRNAs and surface proteins in a solution containing exosomes. Although this method provides a beneficial platform for a simple and non-invasive liquid biopsy for the diagnosis of various diseases, it involves several washing steps that make the entire detection process laborious.

**Figure 1:**
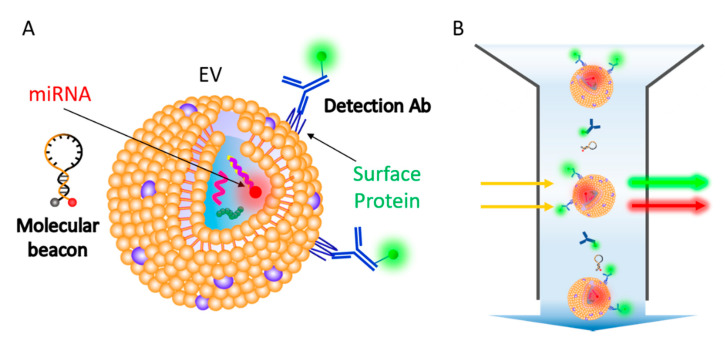
Schematic representation for single step in situ detection of extracellular vesicles (EV) surface proteins and miRNAs using a flow cytometer. (**A**) EV surface proteins and miRNA markers can be simultaneously detected using fluorescent dye-conjugated antibodies and molecular beacons. (**B**) A single step flow cytometric analysis of EV proteins and miRNAs.

In the present study, a flow cytometry-based method was developed for single step in situ detection of EV surface proteins and inner miRNAs (Figure 1A). This method does not require any washing step for detection and measures the fluorescence signals from each EV particle using a flow cytometer (Figure 1B). In addition, to enhance EV biomarker detection using a regular flow cytometer, we also developed a novel method that enabled EV cluster formation using 1,2-distearoyl-sn-glycero-3-phosphoethanolamine (DSPE)-polyethylene glycol (PEG)-DSPE conjugate, which resulted in improved biomarker detection signals. The integrated method does not require any washing step for the detection and measures fluorescence signals by increasing the chance of detection by flow cytometer, which makes it a labor- and cost-saving method with enhanced accuracy. Because cancers are heterogeneous and high-throughput cancer detection is required, the method in this study can provide a much-needed platform for predicting prognosis and monitoring cancer treatment responses in the clinical stage.

## 2. Materials and Methods

### 2.1. Cell Culture and EV Isolation

Human dermal fibroblast (HDF) was a kind gift from Prof. K. M. Park at Incheon National University, Republic of Korea. HeLa cells were purchased from the Korean Collection for Type Cultures (Korea). HDF (normal) and HeLa (cancer) cells were cultured in a humidified atmosphere of 5% CO_2_ at 37 °C in Dulbecco’s Modified Eagle Medium (Corning, Corning, NY, USA) supplemented with 10% (v/v) fetal bovine serum (FBS; Gibco, Gaithersburg, MD, USA) and 1% (v/v) penicillin-streptomycin. For EV production, each cell was cultured in media containing 10% EV-free FBS. EV-free FBS was generated using centrifugation at 120,000× *g* for 10 h at 4 °C with a TLA-100.3 fixed-angle rotor (Optima TL-100; Beckman Coulter, Brea, CA, USA). The supernatant was collected and filtered using a 0.22 µm cellulose acetate syringe filter (GVS, Bologna, Italy) and stored at −80°C until further use. EV isolation was performed using ExoQuick-TC™ EV precipitation solution (System Biosciences, Palo Alto, CA, USA), according to the manufacturer’s instructions. Cell culture media were centrifuged at 3000× *g* for 15 min at 4 °C. After centrifugation, the supernatant was filtered using a 0.22 μm cellulose acetate syringe filter and mixed with ExoQuick-TC™ solution. The mixture was kept at 4 °C overnight and then centrifuged at 1500× *g* for 30 min at 4 °C. The EV pellet was dissolved in 1× PBS and stored at −80 °C until further use.

### 2.2. Quantification of EV Particles and Total Protein Content

The number and sizes of the EVs were measured using nanoparticle tracking analysis (NTA, threshold 4, time 30 s, frame particles <100) with the NanoSight NS300 system (Malvern Panalytical, Malvern, UK). The camera focus was adjusted to visualize sharp individual dots. The total protein concentration was measured using a BCA assay (Thermo Fisher Scientific, Waltham, MA, USA). The BCA working reagent was prepared according to the manufacturer’s protocol. Unknown samples and standards were diluted and mixed with the reagent solution and incubated at 37 °C for 30 min. The absorbance was measured at 562 nm using a spectrophotometer.

### 2.3. Exosomal RNA Isolation, cDNA Synthesis, and Real-Time PCR Analysis

RNA was extracted from the EVs using the FavorPrep™ Tri-RNA Reagent (Favorgen Biotech Corp., Ping-Tung, Taiwan), according to the manufacturer’s protocol. The RNA concentration and purity were evaluated using a NanoDrop™ Lite spectrophotometer (Thermo Fisher Scientific, Waltham, MA, USA). miRNA was reverse-transcribed stem-loop using an miScript RT II Kit (Qiagen, Hilden Germany) followed by real-time PCR amplification using a StepOnePlus™ Real-Time PCR System (Thermo Fisher Scientific, Waltham, MA, USA) with an miScript SYBR^®^ Green PCR Kit (Qiagen, Hilden Germany), which was specific for mature miRNA sequences. U6 small nuclear RNA (snRNA) was used as an internal control for the SYBR^®^ Green miRNA assay to analyze the expression levels of miRNAs in EVs.

### 2.4. Dynamic Light Scattering and Western Blot Analysis

The zeta potential of the EVs was measured using dynamic light scattering with a Malvern Zetasizer Nano ZS (Malvern Panalytical, Malvern, UK) at 25 °C. The same EV concentrations were used to adjust equal volumes for analysis (laser 4 mW, wavelength 633 nm). For Western blot, EVs were lysed in RIPA buffer (Rockland Immunochemicals, Pottstown, PA, USA), following which the protein concentrations were determined using BCA assay. Proteins were separated using sodium dodecyl sulfate-polyacrylamide gel electrophoresis (SDS-PAGE) under reducing (TSG101) or non-reducing (CD63, CD81) conditions. Equal amounts of EVs (20 μg) were used for comparison. A Western blot analysis was performed with primary antibodies, including mouse anti-TSG101 (Abcam, ab83, Cambridge, UK) at 1:1000 dilution, mouse anti-CD63 (MBL International Corporation, MEX002-3, Woburn, MA, USA) at 1:1000 dilution, mouse anti-CD81 antibody (Abcam, ab79559, Cambridge, UK) at 1:1000 dilution, rabbit anti-Syntenin (Abcam, ab133267, Cambridge, UK) at 1:2000, rabbit anti-Hsc70 (Abcam, ab51052, Cambridge, UK) at 1:500, rabbit anti-GM130 (Abcam, ab52649, Cambridge, UK) at 1:1000, and rabbit anti-calnexin (Cell Signaling Technology, 2679S, Danvers, MA, USA) at 1:1000. For detection of the proteins, a horseradish peroxidase-conjugated anti-mouse secondary antibody (Abcam, ab6728, Cambridge, UK) at 1:2000 dilution, and anti-rabbit IgG (Cell Signaling Technology, 7074S, Danvers, MA, USA) at 1:1000 and an enhanced chemiluminescence detection system (Bio-Rad, Hercules, CA, USA) were used. Images were captured using ChemiDoc™ XRS+ imaging system (Bio-Rad, Hercules, CA, USA).

### 2.5. miRNA Detection Using Molecular Beacon

A MB was designed to target mature miR-21 (MB-21), with the sequence 5′-Cy5-GCGCGTCAACATCAGTCTGATAAGCTACGCGC-BHQ2-3′ [20,21,22,23]. MB-21 with cyanine-5 (Cy5) was designed using the UNAFold tool provided by Integrated DNA Technologies (IDT, Coralville, IA, USA) and synthesized by IDT. The specificity of the MB was evaluated by mixing MB-21 and synthetic miR-21 or EVs and incubating at 37 °C for 1 h. Hybridization of MB-21 with the synthetic miR-21 or with miR-21 in EVs was assessed using a Varioskan™ Flash Multimode Reader (Thermo Fisher Scientific, Waltham, MA, USA) at excitation and emission wavelengths of 650 nm and 670 nm, respectively.

### 2.6. Flow Cytometric Analysis of EV CD63 and miR-21 Detection and Induction of EV Cluster Using DSPE-PEG-DSPE

EVs from normal and cancer cells were incubated at 37 °C for 1 h with Alexa Fluor^®^ 488-conjugated CD63-targeting antibody [Thermo Fisher Scientific, USA; in a 1:50 volume ratio (antibody:solution)] for EV surface CD63 detection and with 100 nM MB for EV miRNA detection. The fluorescence signals were analyzed using Varioskan™ Flash Multimode Reader in a 384-well plate (Corning, Corning, NY, USA) or CytoFLEX (Beckman Coulter, Brea, CA, USA) in a microcentrifuge (SPL Life Sciences, Gyeonggi-do, Korea). For accurate analysis, a QC was performed through calibration at least once every 2 weeks using CytoFLEX Daily QC Fluorospheres (Beckman Coulter, Brea, CA, USA). For the detection of EVs using CytoFLEX, the sample flow rate was adjusted to slow (10 µL/min) [24,25]. The stop criterion was set for a time at 300 s or the events 10,000 [26]. The data were analyzed using CytExpert (Beckman Coulter, Brea, CA, USA). The fluorescence signals were assessed at an excitation wavelength of 495 nm and an emission wavelength of 519 nm for CD63-targeting antibody, and an excitation wavelength of 650 nm and an emission wavelength of 670 nm for MB-21.

To detect biomarkers in EV clusters, the different concentrations of DSPE-PEG-DSPE (0.25–4 mg/mL) were tested to find the optimal DSPE-PEG-DSPE concentration (0.5 mg/mL). DSPE-PEG-DSPE (Biochempeg Scientific Inc., Watertown, MA, USA), 100 nM MB, and 1:50 volume ratio of antibody was incubated together with 1 × 10^10^ EVs at 37 °C for 2 h. PEG with a molecular weight of 10 kDa was used for this reaction. The fluorescence signals were observed using a fluorometer or a flow cytometer.

### 2.7. Statistical Analysis

Statistical analysis to assess differences between 2 groups was performed using a paired *t*-test with GraphPad Prism 7 software (GraphPad Software, San Diego, CA, USA).

## 3. Results

### 3.1. Characterization of EVs from Cancerous and Non-Cancerous Cells

**Figure 2:**
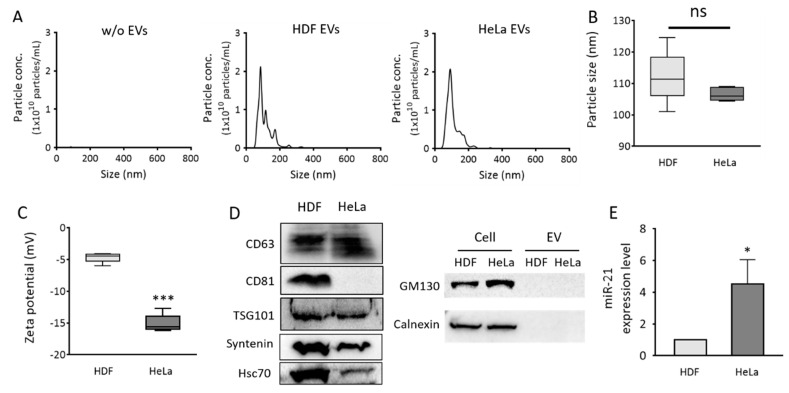
Size distribution, zeta potentials, protein markers, and miR-21 expression levels of EVs from normal and cancer cells. Normal (HDF) and cancer (HeLa) cell-derived EVs were purified using the ExoQuick-TC™ precipitation solution. (**A**) Size distributions of particles isolated from HDF (middle) and HeLa (right) cells were analyzed using nanoparticle tracking analysis (NTA). PBS solution without EVs was measured as control (**B**) Average sizes of particles. (**C**) Zeta potentials of normal and cancer EVs. (**D**) Western blot analysis of EV protein markers, CD63, CD81, TSG101, Syntenin, and Hsc70, and non-EV protein markers, GM130 and calnexin; 20 μg EV proteins were loaded to each lane for detection. (**E**) Real-time PCR analysis was performed to quantify relative EV miR-21 levels in normal and cancer EVs. The miR-21 levels were normalized to those of U6 snRNA. All values are expressed as mean ± SD (* *p* < 0.05, *** *p* < 0.001; ns: Not significant; *n* = 3).

To develop a single step in situ detection method for EV surface proteins and inner miRNAs using a flow cytometer, HDF-derived EVs (normal EVs) and HeLa cell-derived EVs (cancer EVs) were first characterized. To begin with, the size distributions and concentrations of the EVs were analyzed using NTA (Figure 2A). The average sizes of the EVs from HDF and HeLa cells were 112 and 106 nm, respectively (Figure 2B). The zeta potentials of normal and cancer EVs were −4.68 and −15.08 mV, respectively (Figure 2C), which indicated that both the EVs were slightly negatively charged. This result was consistent with a previous report that the zeta potential of cancer EVs was more negatively charged than that of normal EVs [27]. Because ExoQuick-TC was used for EV isolation and the EV purity was not examined, the results analyzed using NTA may contain the contaminants.

Western blot for EV markers, including CD63, CD81, TSG101, Syntenin, and Hsc70, and non-EV markers, including GM130 and calnexin, demonstrated that most of the isolated particles were EVs (Figure 2D). HeLa EVs had much lower levels of CD81 than HDF EVs. CD63 levels in HeLa EVs were slightly higher than those in HDF EVs, and it was chosen as the representative EV surface protein marker for the flow cytometric detection of EV markers in this study. miR-21 levels in each set of EVs were also analyzed using real-time PCR. As expected, cancer EVs displayed 4.5-fold higher miR-21 levels than normal cell-derived EVs (Figure 2E). This result was consistent with data from previous reports [28,29], indicating that EV miR-21 can be used as a potential cancer diagnostic biomarker. Accordingly, miR-21 was chosen as an EV miRNA marker for flow cytometric detection in this study.

### 3.2. In Situ Detection of miR-21 in EVs Using MB

**Figure 3:**
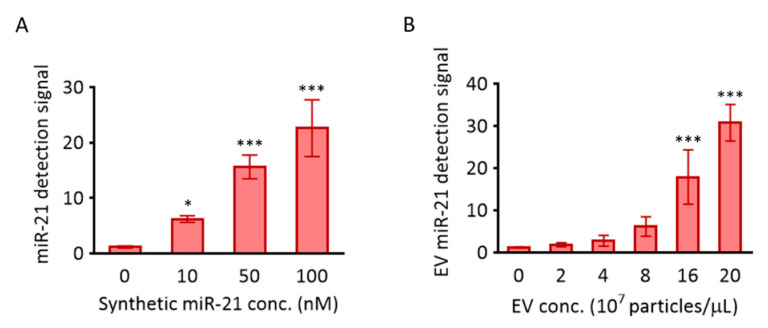
Detection of miR-21 using MB-21. The molecular beacon designed to target miR-21 was investigated for its hybridization to miR-21 in solution (**A**) and cancer EVs (**B**). (**A**) 100 nM MB-21 was incubated with different concentrations of synthetic miR-21 (0–100 nM), followed by measurement of the fluorescence signal using a fluorometer. (**B**) 100 nM of MB-21 was incubated with different concentrations of HeLa cell-derived EVs (0–20 × 10^7^ particles/µL). All values are expressed as mean ± SD (* *p* < 0.05, *** *p* < 0.001; *n* = 3–6).

MB-21, designed for miR-21 detection, was tested to assess its hybridization property with various concentrations of the target in solution. There was a gradual, significant, and miR-21 concentration-dependent increase in the corresponding fluorescence intensities from MB-21 (Figure 3A). For instance, the fluorescence intensity increased by 13.4-fold when the target miR-21 concentration increased from 0 to 100 nM. Based on this result, MB-21 was further tested for in situ hybridization with miR-21 in EVs (Figure 3B). Different concentrations of EVs (0–20 × 10^7^ particles/µL) were incubated with MB-21 and the resultant detection signals were observed. The fluorescence signal from MB-21 incubated without EVs was very low. The signal increased drastically upon incubation of MB-21 and EVs together, and kept increasing gradually as the concentration of the EVs increased. Thus, in situ detection of EV miR-21 was successfully achieved using MB.

In addition, there are non-EV associated miRNAs that can interfere with miRNA detection. In this case, the miRNA-protein complex should be removed by treating the EV sample with protease/RNase. However, in situ EV miRNA detection using MB does not require this pretreatment because of a steric hindrance provided by the associated protein that MB cannot hybridize with miRNA. We have previously compared the miRNA detection signals of MB between non-treated and RNase-treated EVs [23], and, as a result, no significant difference in the detection signal was observed.

### 3.3. Fluorometer-Based Detection of EV miRNA and Surface Protein

EVs are nano-sized particles that cannot be isolated or washed using regular centrifugation. Ultracentrifugation is accompanied by a significant loss of EVs during centrifugation, and thus, it is not considered suitable for diagnostic purposes. Polymer-based precipitation methods have been criticized for their co-precipitation of impurities, including proteins. Thus, it is necessary to develop a method for in situ detection of EV biomarkers for high-throughput liquid biopsy or point-of-care testing using EV biomarkers. There is a need to develop techniques for simultaneous detection of various types of EV biomarkers to achieve highly accurate and specific diagnostic methods. The main purpose of the method is to develop the high-throughput disease diagnosis by in situ EV biomarker detection using a flow cytometer. One of the advantages of the simultaneous detection of EV surface protein and miRNA using a flow cytometer is that it can distinguish the EVs from the contaminants. For instance, it is unlikely that the contaminant has both EV surface protein and miRNA at the same time. In addition, the sizes of protein contaminants are usually smaller than those of EVs that they can hardly be detected by a flow cytometer.

**Figure 4:**
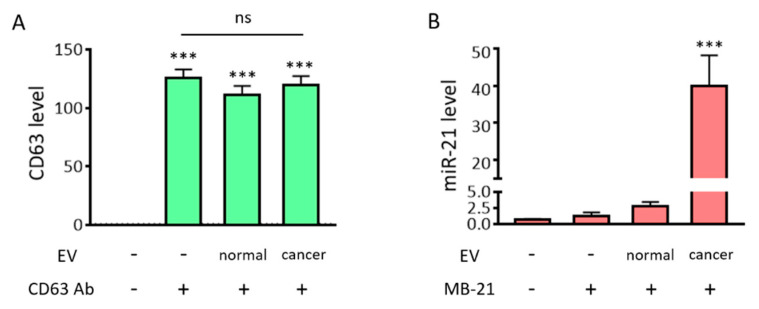
Fluorometer-based detection of EV miRNAs and surface protein markers. Simultaneous in situ detection of EV surface protein (CD63) and miRNA (miR-21) was tested. Fluorescent signals were assessed using a fluorometer. (**A**) Fluorescent intensities from the fluorescence dye labelled-CD63 antibody, in the absence/presence of EVs, were assessed. There was a drastic increase in the fluorescent signals from the CD63 antibody, regardless of the presence/absence of EVs and type, which suggests that the unbound free CD63 antibody must be removed for specific EV CD63 detection. (**B**) The fluorescent intensities from MB-21, in the absence/presence of EVs, were assessed. There was no significant difference in fluorescent signals upon addition of MB-21 in the absence of EVs. This could be attributed to the self-quenching ability of MB in the absence of target miRNA. The detection signal increased when MB-21 was incubated with normal EVs, and further increased upon incubation of MB-21 with cancer EVs. All values are expressed as mean ± SD (*** *p* < 0.001; ns: Not significant; *n* = 3–4).

To evaluate the simultaneous in situ detection of miR-21 and protein from EVs using MB and fluorescence dye-labeled antibodies, antibodies targeting CD63 and MB-21 were incubated in the absence or presence of EVs (2 × 10^8^ particles/µL), following which the fluorescent signals obtained were analyzed using a fluorometer. As shown in Figure 4A and as expected, high fluorescent signals were observed, regardless of EV presence and type. This indicated that the CD63-targeting free antibodies that did not bind to CD63 on the EV surface still contributed to the detection signal because the antibody did not have a fluorescence quencher. In contrast, there was no noticeable increase in the fluorescence intensity when MB-21 was incubated without EVs, because MB had self-quenching ability due to the presence of a quencher (Figure 4B). The detection signals increased by 2.4-fold when MB-21 was incubated with HDF cell-derived EVs. The real-time PCR results shown in Figure 2E suggested that the normal EVs also contained miR-21. When MB-21 was incubated with cancer EVs derived from HeLa cells, the fluorescence intensity further increased 34-fold, consistent with the real-time PCR results. Thus, we can infer from these results that free unbound protein-targeting antibodies significantly interfere with the detection of EV surface biomarkers. In our previous studies on simultaneous detection of miRNAs inside EVs and proteins on EV surfaces, the EVs were captured using magnetic beads for the separation of unbound probes. However, in this method, it was still necessary to wash and remove the unbound probes to distinguish the detection signals from the background signals. Accordingly, the method developed in the current study, which does not require a washing process for the removal of the unbound probe, was beneficial for EV biomarker detection.

### 3.4. Single Step In Situ Flow Cytometric Detection of EV Surface Protein and miRNA

We have previously developed in situ multiplexed detection of EV biomarkers using magnetic beads [20]. The method used a fluorometer to quantify the fluorescent intensities of the miRNAs and surface proteins in a solution containing EVs. First, EVs that do not get captured have to be removed with a washing step. In addition, unlike MB, which has a self-quenching ability due to the presence of a quencher on one side, fluorescence signals from the free unbound antibody have to be eliminated, as the antibody has no self-quenching activity. These additional washing steps make the entire detection process laborious and may result in a loss of the captured EVs, and in turn, a loss of the detection signals. Moreover, quantitative measurement of average fluorescence signals using a fluorometer may result in under- or over-estimation, since only a part of the population is used for the measurement. Consequently, it is necessary to develop a method that can discriminate between bound and unbound probes to achieve single step detection of EV biomarkers. Thus, flow cytometric analysis was introduced because the method can be used without removing free probes, especially for cellular biomolecule analysis. Despite the recent developments in flow cytometers, they still have a limitation in terms of analyzing nanoscale particles, including EVs.

**Figure 5:**
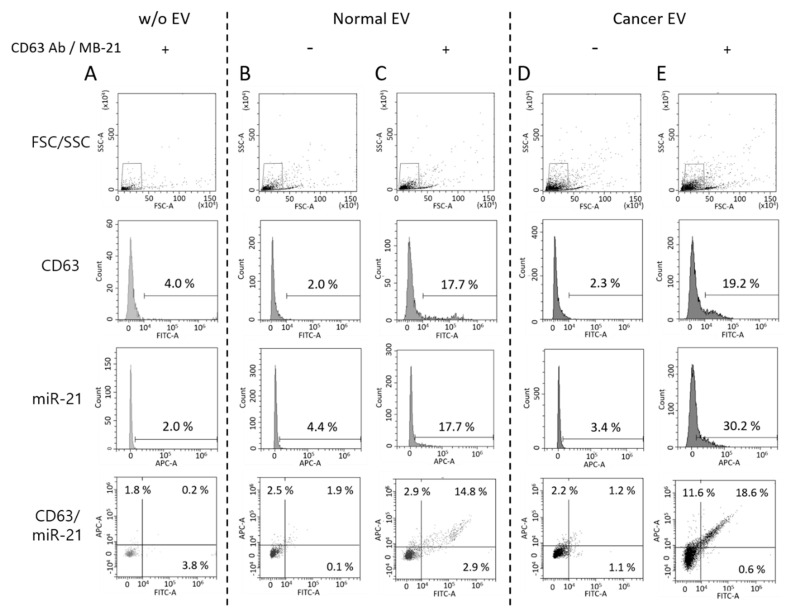
Single step detection of EV surface proteins and miRNAs using flow cytometry. Flow cytometric analysis was performed for simultaneous detection of EV CD63 and miR-21 in a single step. Forward versus side scatter (FSC/SSC) gatings are shown in the top panel. (**A**) Representative flow cytometric results for non-EV control with CD63 antibody and MB-21 (**B**,**C**) Representative flow cytometric results for normal EV biomarker detection without (**B**) and with (**C**) CD63 antibody and MB-21. CD63 and miR-21 fluorescent signals from normal EVs without detection probes were used as background controls. The numbers in the quadrant plot of CD63 (x-axis) and miR-21 (y-axis) represent the percentages of each population, which indicated that there was an increase in the number of CD63 and miR-21 positive EVs. (**D**,**E**) Single step in situ detection of CD63 and miR-21 in cancer EVs. Fluorescent signals were observed for cancer EVs in the absence (**D**) and presence (**E**) of CD63 antibody and MB-21. There was an increase in the percentages of miR-21 positive EVs in the cancer EVs, as compared to that in the normal EVs. As a result, a higher population of EVs positive for both CD63 and miR-21 was observed.

In this context, we also tested if EVs labeled with antibody and MB could be visualized using a flow cytometer. Therefore, we tested a simultaneous and single step in situ flow cytometric detection of EV protein and miRNA using a combination of antibody and MB. First, we checked that free CD63 antibody and MB-21 in the absence of EVs were not detected by a flow cytometer (Figure 5A). Then, we analyzed the normal EVs produced from HDF in the absence (Figure 5B) and presence (Figure 5C) of CD63 antibody and MB-21. The fluorescent signals for both CD63 and miR-21 increased when CD63 antibody and MB-21 were added to normal EVs, indicating that CD63 antibody and MB-21 can detect CD63 on the surface and miR-21 inside of EVs, respectively. The CD63 positive population increased from 2.0% to 17.7%, while the miR-21 positive population increased from 4.4% to 17.7% in normal EVs, as clearly seen in the quadrant plots. There are lots of particles in the main peak, meaning that the levels of CD63 or miR-21 were relatively low or the size of EVs was so small that they could not be measured or passed through without being detected by the flow cytometer. However, since there were still many EVs that were recognized and detected by the flow cytometer, it was noteworthy that EVs stained with antibodies targeting CD63 could be detected without any washing step.

In the next stage, a single step in situ detection of CD63 and miR-21 in cancer EVs produced from HeLa cells was tested using flow cytometry (Figure 5D,E). Once again, the cancer EVs alone did not show a significant increase in the fluorescent signals for both CD63 (2.3%) and miR-21 (3.4%) (Figure 5D). However, when CD63 antibody and MB-21 were added to the cancer EVs, followed by flow cytometric analysis, the CD63 positive EVs increased to 19.2%, while the miR-21 positive EVs among cancer EVs increased to 30.2%. The differences in the molecular profiles of EVs can be clearly seen in the quadrant of the flow histogram. That is, although CD63 positive EVs (x-axis) in a sub-population were almost the same between normal and cancer EVs, cancer-related miRNA, miR-21 positive EVs (y-axis) increased in cancer EVs. Thus, we can infer from this result that a single step in situ detection of EV surface proteins and inner miRNAs can be achieved using flow cytometry.

### 3.5. Induction of EV Clustering Using DSPE-PEG-DSPE

Although we have demonstrated that EV proteins and miRNAs can be detected in a single process using flow cytometry, there were still some undetected EVs among the total population. This was probably due to the nanoscale size of EVs. As mentioned earlier, the flow cytometer commonly used for cell analysis had limitations in terms of the detection of nanoscale particles. A high-resolution flow cytometer may provide better performance in the detection of EVs. However, because the purpose of this method was to analyze EV biomarkers using a regular flow cytometer, increasing the chances of detecting EVs may in turn greatly enhance the efficiency and accuracy of in situ EV biomarker detection using flow cytometry.

**Figure 6:**
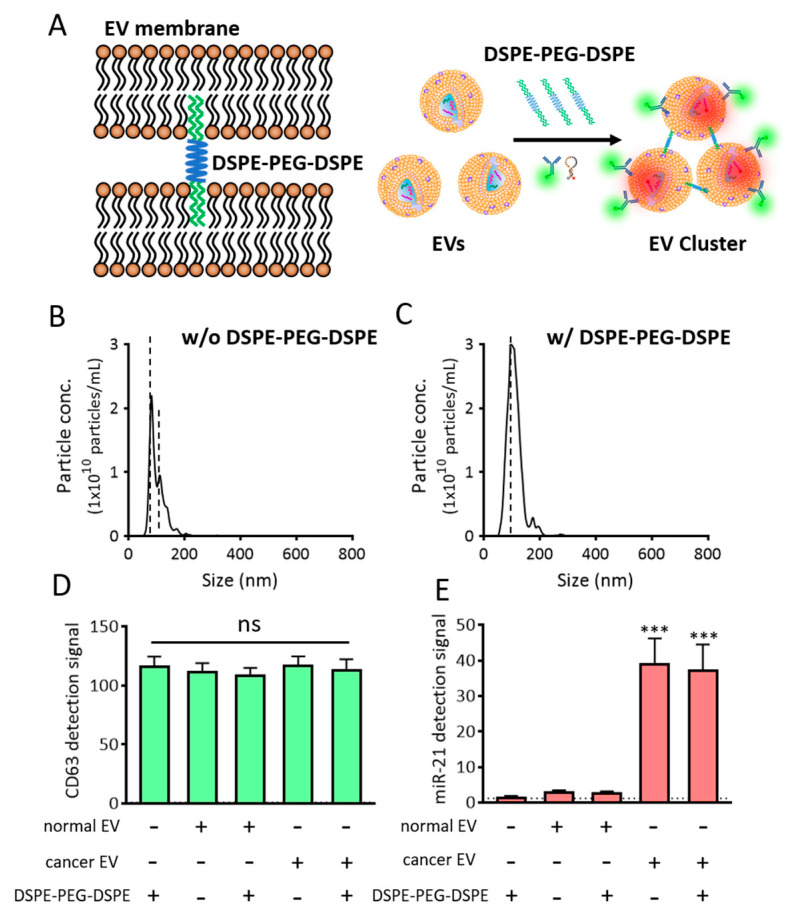
EV cluster formation using DSPE-PEG-DSPE. (**A**) Schematic representation of DSPE-PEG-DSPE-induced EV cluster formation and single step detection of EV biomarkers in clustered EVs using flow cytometry. (**B**,**C**) NTA analysis of cancer particle size distribution in the absence (**B**) and presence (**C**) of DSPE-PEG-DSPE. Note that the EV clusters were observed to be larger than single EVs. (**D**,**E**) Effects of DSPE-PEG-DSPE on the fluorescent signals for CD63 (**D**) and miR-21 (**E**), as assessed using a fluorometer. All values are expressed as mean ± SD (*** *p* < 0.001; ns: Not significant; *n* = 3–6).

To accomplish this, we developed a method that induced clustering of EVs using a phospholipid-polymer-phospholipid conjugate, DSPE-PEG-DSPE (Figure 6A). DSPE is a phospholipid in this conjugate, while PEG is used as a linker [30,31,32]. We assumed that the DSPE on one side of the DSPE-PEG-DSPE conjugate can capture one EV owing to its lipid solubility, while the DSPE on the other side can capture another EV. This may enhance the detection of EV biomarkers using regular flow cytometry, as the DSPE-PEG-DSPE conjugate will assemble multiple EVs to form clusters, thereby enlarging their overall size. To test our hypothesis, EVs were incubated with 0.5 mg/mL of DSPE-PEG-DSPE for 2 h, after which, the particle sizes of the EVs before and after DSPE-PEG-DSPE treatment were assessed using NTA (Figure 6B,C). It was noteworthy that upon treatment with DSPE-PEG-DSPE, we observed clusters with sizes larger than those of single EVs. The average size increased from 100 to 110 nm upon DSPE-PEG-DSPE treatment, while the size of the main peak increased from 82 to 101 nm. Subsequently, it was predicted that the increased size of the EV cluster upon DSPE-PEG-DSPE treatment could contribute to enhanced simultaneous in situ detection of EV biomarkers. The NTA itself has a systematic error that possibly displays fluctuations. Thus, further investigation was required to directly demonstrate EV cluster and the average number of EVs in the cluster using a transmission electron microscope.

Before further optimizing the biomarker detection from these EV clusters, we checked whether DSPE-PEG-DSPE itself affected or interfered with the fluorescent signals (Figure 6D,E). When DSPE-PEG-DSPE was incubated with CD63-targeting antibody (Figure 6D) or MB-21 (Figure 6E), no changes were observed in the fluorescence intensities, which indicated that DSPE-PEG-DSPE did not interact and cluster the antibody and MB. In addition, normal and cancer EVs were incubated with DSPE-PEG-DSPE in the presence of detection probes, following which the overall fluorescence intensities were assessed using a fluorometer. There were no noticeable changes in the fluorescent signals, indicating that DSPE-PEG-DSPE did not affect the fluorescent signals in fluorometer-based biomarker detection.

### 3.6. Single Step In Situ Detection of Protein and miRNA in EVs Clustered Using DSPE-PEG-DSPE

To accomplish the detection of proteins and miRNAs in EVs in a single step, DSPE-PEG-DSPE was co-incubated for 2 h with the EVs, in the presence of CD63 antibody and MB-21, followed by flow cytometric analysis of the obtained fluorescence signals. DSPE-PEG-DSPE can form the micelles, and these self-assembled structures can affect the detection signals. To investigate this, DSPE-PEG-DSPE alone was incubated with CD63 antibody and MB-21 in the absence of EVs.

**Figure 7:**
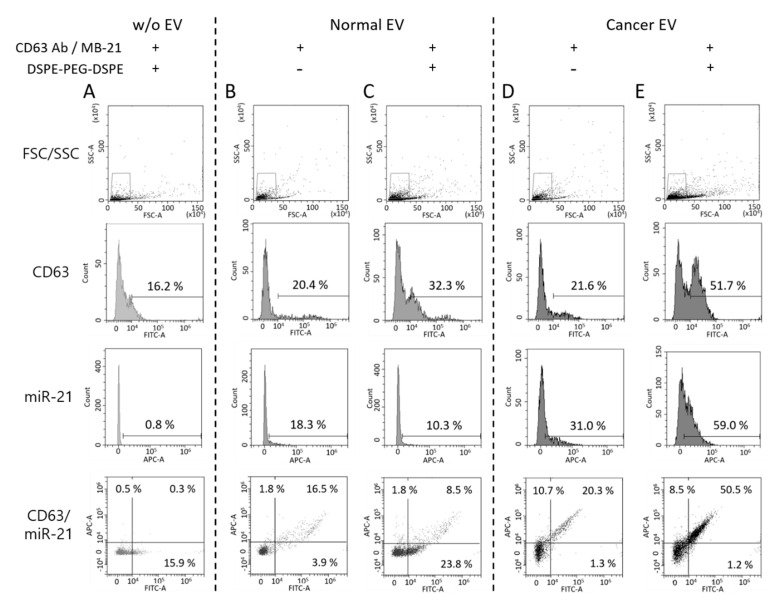
Single step in situ detection of proteins and miRNAs in clustered EVs using a flow cytometer. Forward versus side scatter (FSC/SSC) gatings are shown in the top panel. (**A**) DSPE-PEG-DSPE alone was incubated with CD63 antibody and MB-21 in the absence of EVs (**B**,**C**) Normal EVs were incubated with CD63 antibody and MB-21 in the absence (**B**) or presence (**C**) of DSPE-PEG-DSPE. The percentages of CD63 and miR-21 positive populations were analyzed using a flow cytometer. The percentages in the quadrant plot of CD63 (x-axis) and miR-21 (y-axis) indicate the number of CD63 and/or miR-21 positive EVs among the total EVs. (D-E) Cancer EVs were analyzed after incubation with CD63 antibody and MB-21 in the absence (**D**) or presence (**E**) of DSPE-PEG-DSPE. The CD63 and miR-21 positive populations were analyzed using a flow cytometer. The quadrant plot of CD63 (x-axis) and miR-21 (y-axis) demonstrates that induction of an EV cluster enhanced the detection efficiency of EV biomarkers using flow cytometry. The overall percentage of CD63 and miR-21 positive EVs increased upon DSPE-PEG-DSPE treatment. Cancer EVs displayed a drastic increase in the populations of both CD63 and miR-21 positive EVs, as compared to normal EVs.

As shown in Figure 7A, CD63 and miR-21 positive populations were only 16.2% and 0.8%, respectively. Thus, the self-assembly of DSPE-PEG-DSPE did not affect the detection using the flow cytometer. Then, normal EVs, co-incubated with CD63 antibody, MB-21, and DSPE-PEG-DSPE were analyzed using flow cytometry (Figure 7B,C). The percentages of CD63 positive EVs were 20.4% and 32.3% in the absence and presence of DSPE-PEG-DSPE, respectively, indicating that there was an increase in the CD63 detection signal upon EV cluster formation. However, the miR-21 positive EVs showed no such increase. This could be because the intrinsic miR-21 level in the normal cell-derived EVs was low and not enough to be visualized even after DSPE-PEG-DSPE treatment. Moreover, the composition of stiffness among EVs originated from different cells can affect the EV cluster formation. Further exploration is needed to elucidate the effect of EV membrane properties on DSPE-PEG-DSPE membrane insertion and subsequent EV clustering. Quadrants were set to delineate between CD63 positive, miR-21 positive, and negative EVs based on this result. Normal EVs were clustered using DSPE-PEG-DSPE, following which each biomarker was simultaneously detected with CD63 antibody and MB-21.

For single step biomarker detection in the cancer cell-derived EV cluster, EVs from HeLa cells were incubated with CD63 antibody and MB-21, in the absence or presence of DSPE-PEG-DSPE (Figure 7D,E). As shown before, in the absence of DSPE-PEG-DSPE, the numbers of both CD63 positive (21.6%) and miR-21 positive (31.0%) EVs were higher in the cancer cells, as compared to those in normal cell-derived EVs. Furthermore, when cancer EVs were clustered using DSPE-PEG-DSPE, the percentages of CD63 positive and miR-21 positive EVs drastically increased to 51.7% and 59.0%, respectively (Figure 7E). This result can be clearly seen in the quadrant flow histogram, which shows that the EV population positive for both CD63 and miR-21 was 50.5%. Considering that the CD63 and miR-21 positive EV populations were only 8.5% (Figure 7C) and 20.3% (Figure 7D) in clustered normal and un-clustered cancer EVs, respectively, we concluded that the novel method for single step in situ detection of protein and miRNA biomarkers in EVs using flow cytometry can be significantly improved upon clustering the EVs using DSPE-PEG-DSPE.

**Figure 8:**
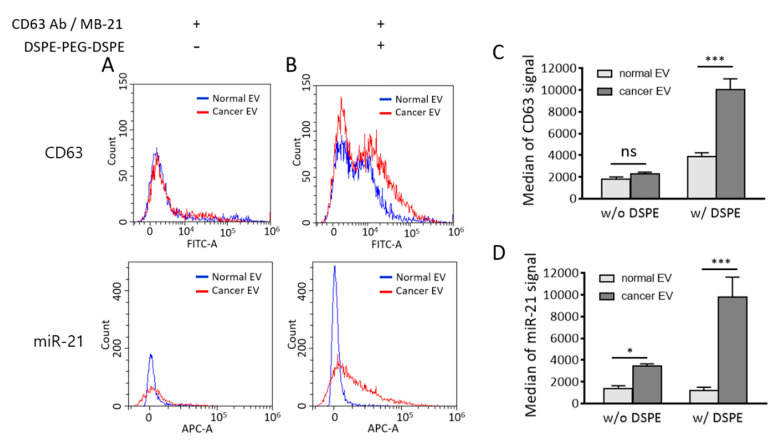
Comparison of flow cytometric results between normal and cancer EVs in the absence and presence of DSPE-PEG-DSPE. (**A**,**B**) Merged flow cytometric results of CD63 (upper) and miR-21 (lower) detection in normal and cancer EVs in the absence (**A**) and presence (**B**) of DSPE-PEG-DSPE. (**C**,**D**) Median fluorescence intensities of CD63 (**C**) and miR-21 (**D**) were calculated from flow cytometric results. All values are expressed as mean ± SD (* *p* < 0.05, *** *p* < 0.001; ns: Not significant; *n* = 3).

The effect of DSPE-PEG-DSPE can be observed in the merged result of flow cytometric analysis between normal and cancer EVs (Figure 8A,B). The median fluorescence signals from CD63 and miR-21 detection were calculated, and they significantly increased upon cluster formation (Figure 8C,D). It is noteworthy that the substantial amount of EV biomarker positive population increased considering the relatively small change of particle size after DSPE-PEG-DSPE treatment. It is possible that the increased size was within the range of the resolution size threshold of a flow cytometer. Further analysis should be followed to elucidate the size-dependent detection property of the flow cytometer.

There is a possibility that DSPE-PEG-DSPE can affect the EV membrane permeability; thereby, more MB-21 can be delivered into EVs. This subsequently resulted in an increased miR-21 positive cancer EV population. There is a possibility that DSPE insertion may affect the membrane property of EVs. An increased MB number entered the EVs may provide more chances of MB-miRNA hybridization and thereby increase detection signals. If this was the case, the fluorescent intensity of MB-21 in cancer EV cluster should also be increased as compared to non-clustered cancer EVs. However, as shown in Figure 6E, miR-21 levels detected by MB-21 were similar in cancer EVs regardless of DSPE-PEG-DSPE treatment analyzed by a fluorometer. This indicated that the increase in the miR-21 positive population of DSPE-PEG-DSPE-treated cancer EVs analyzed by flow cytometer was not originated from the effect of DSPE-PEG-DSPE on EV membrane permeability.

Previously developed multiplexed EV biomarker detection method using captured EV onto magnetic beads has several limitations. First, the detection process requires multiple manipulations that are laborious and time-consuming. Thus, it may not be suitable for high-throughput diagnosis of disease. In addition, due to the low EV capturing efficiency, many EVs were lost during sample preparation. The magnetic bead made of iron oxide may interfere with the detection signal by quenching the fluorescence due to a magnetic bead and EVs’ proximity. Further analysis is required to directly demonstrate the formation of EV cluster using DSPE-PEG-DSPE. In addition, the method should be adjusted and optimized to the clinical samples, including human serum and urine. Overall, multiplexed in situ detection of EV protein and miRNA using a flow cytometer without magnetic beads can overcome those limitations. EV cluster formation using DSPE-PEG-DSPE has not been reported before, and this is the first report investigating the flow cytometric detection of multiple EV biomarkers using EV cluster formation.

## 4. Conclusions

EVs are nanoparticles that are difficult to handle; thus, it is essential to develop a method that enables efficient and straightforward EV biomarker detection. In addition, there is a need to develop a technique for multiplexed biomarker detection in a single EV to increase the value of using EVs over other circulating biomarkers in liquid biopsy. In the present study, we present a technique for a simultaneous single step in situ detection of EV surface proteins and inner miRNAs using a flow cytometer. To improve the detection resolution of flow cytometry, we used DSPE-PEG-DSPE to induce clustering of the EVs, which enhanced the detection signals produced in the presence of the detection antibody and MB. Thus, a single step in situ detection of multiple EV biomarkers using a flow cytometer can be developed as a simple, labor- and time-saving, non-invasive liquid biopsy for the diagnosis of various diseases (including cancer) that can provide a much-needed platform to predict the prognosis and monitor treatment responses in the clinical stage.
